# De novo Balanced Robertsonian Translocation rob(22;22)(q10;q10) in a Woman with Recurrent Pregnancy Loss: A Rare Case

**Published:** 2018

**Authors:** Nawras Alhalabi, Walid Al-Achkar, Abdulsamad Wafa, Mazen Kenj, Marwan Alhalabi

**Affiliations:** 1- Faculty of Medicine, Syrian Private University, Damascus, Syria; 2- Department of Molecular Biology and Biotechnology, Human Genetics Division, Atomic Energy Commission of Syria, Damascus, Syria; 3- Assisted Reproduction Unit, Orient Hospital, Damascus, Syria; 4- Kenj Cytogenetics Laboratory, Damascus, Syria; 5- Department of Reproductive Medicine, Genetics and Embryology, Faculty of Medicine of Damascus University, Damascus, Syria

**Keywords:** Assisted Reproduction Techniques, Recurrent Pregnancy Loss, Robertsonian translocation, Syria

## Abstract

**Background:**

Recurrent pregnancy loss (RPL), one of the most common complications of pregnancy, is responsible for significant emotional distress to the couple desiring to conceive. In almost 50% of the cases, the etiology remains unknown. The frequency of chromosomal structural rearrangements associated with a history of RPL in couples varies between 2% to 8%. Robertsonian translocations (ROBs) have an estimated incidence rate of 1/1000 births, making this type of rearrangement the most common structural chromosomal abnormalities seen in the general population. According to the literature, there are few RPL cases with rob (22;22).

**Case Presentation:**

This case is a Syrian female offered to the Orient Hospital (Damascus, Syria), having RPL in the first trimester, no fetal malformations, and/or no neonatal death. She had a balanced chromosomal translocation involved the both short arms of chromosome 22. Banding cytogenetics, refined by array-proven multicolor banding (aMCB) revealed a rob (22; 22)(q10;q10). Her husband had a normal karyotype. Interestingly, chromosomal analysis was performed for her other family members and it revealed normal karyotype for all people, which indicates that translocation is of de novo origin. However, the couple did not have any living offspring after seven years of marriage.

**Conclusion:**

The present case was a case of RPL occurring due to rob (22;22). However, the rob(22;22)(q10;10) is the cause of recurrent abortions. Couples with the history of RPL should be suggested to do cytogenetic analysis in order to estimate whether they have chromosomal rearrangement. This diagnostic approach is of great significance to figure out what causes RPL.

## Introduction

Recurrent pregnancy loss (RPL), one of the most common complications of pregnancy is responsible for significant emotional distress to the couple desiring to conceive. RPL is defined as the occurrence of two or more consecutive abortions and it affects about 1–5% of couples trying to establish a family (1–3). About 10 to 15% of the clinically recognizable pregnancies result in pregnancy loss, with an additional pre-clinical loss of 22% (4, 5). Determining the cause of a pregnancy loss is important to determine whether further interventions are necessary, as well as to provide a sense of closure to the patient and her partner.

However, in almost 50% of the cases, the etiology remains unknown. Several factors have been suggested to be involved including endocrine dysfunction, autoimmunity, genetic abnormalities, advanced maternal and paternal age, infectious diseases, environmental toxins, congenital and structural uterine anomalies and more (6, 7). Transmission of parental chromosomal abnormalities may be one of the causes for RPL in the first trimester of pregnancy (8, 9). The frequency of chromosomal structural rearrangements associated with a history of RPL in couples varies between 2% to 8% (4, 10–12), which is higher than the general population frequency of 0.7% (10). Robertsonian and reciprocal translocations are more commonly implicated compared to inversions (10, 12–14).

In this paper, a rare case of de novo balanced ROB was reported involving both short arms of chromosomes 22 in a Syrian female with a history of RPL.

## Case Presentation

In Damascus, on September 2013, a 29-year-old, non-smoker Syrian female presented to the fertility clinic, Orient Hospital, due to recurrent pregnancy losses. She reported five miscarriages with the last one occurring two years ago. She had been married for 7 years, has regular menses and her first menarche was at 12 years of age. Her surgical history included two curettage aspirations and a hysteroscopy in 2010 with normal findings. BMI (body mass index) was 22.1. Physical examination was within normal limits, pelvic ultra sonography revealed normal findings which were confirmed by hysterosalpingography. Hormones profile included thyroid-stimulating hormone (TSH) 1.17 *mIU/L* (0.5–4.5 *mIU/L*), free thyroxine (free T_4_) 1.30 *ng/dl* (0.80–1.80 *ng/dl*), follicle stimulating hormone (FSH) 6.5 *mmol/ml* (3.4–10 *mmol/ml*), luteinized hormone (LH) 5.4 *mmol/ml* (1.6–8.3 *mmol/ml*), prolactin (PRL) 17 *ng/ml* (3.6–20 *ng/ml*), estradiol (E2) 35 *pg/ml* (up to 50 *pg/ml*) (all within normal limits). Thrombophilia workup revealed homocysteine 9 μ*mol/L* (5–12 *umol/L*), activated protein C resistance 197 sec (120–400 sec), anticardiolipin IgG antibodies 4 *U/ml* (up to 10 *U/ml*), anticardiolipin IgM antibodies 2 *U/ml* (up to 10 *U/ml*), antithyroid peroxidase (anti-TPO) 14 *IU/ml* (up to 35 *IU/ml*), antithyroglobulin antibodies19.8 *IU/ml* (up to 40 *IU/ml*) and lupus anti-coagulant was also negative. Immunological tests for anti-toxoplasmosis IgG antibodies were 199 *IU/ml* (up to 8 *IU/ml*) with an anti-toxoplasmosis IgM antibodies index of 4.1, indicating past infection with immunity. Anti-rubella IgG antibodies were 102 *IU/ml* (up to 10 *IU/ml*) and anti-rubella IgM antibodies index of 0.4 *IU/ml* (up to 10 *IU/ml*), which also signifies past infection with immunity. Although the patient was advised not to get pregnant, on December 2013, a gestational sac was noted on ultrasound. On January 2014, the conceptus was arrested at 6 weeks of pregnancy.

Her husband (38 years old), a smoker, had a BMI of 23.4. His semen analysis showed normal parameters according to world health organization criteria of 2010 (15). The couple was healthy and phenotypically normal and they were referred for chromosomal analysis based on these findings. A written informed consent was obtained from the couple before writing this report. The Institution Ethical Committee approved the report and the approval is available upon request.

**Figure 1.**
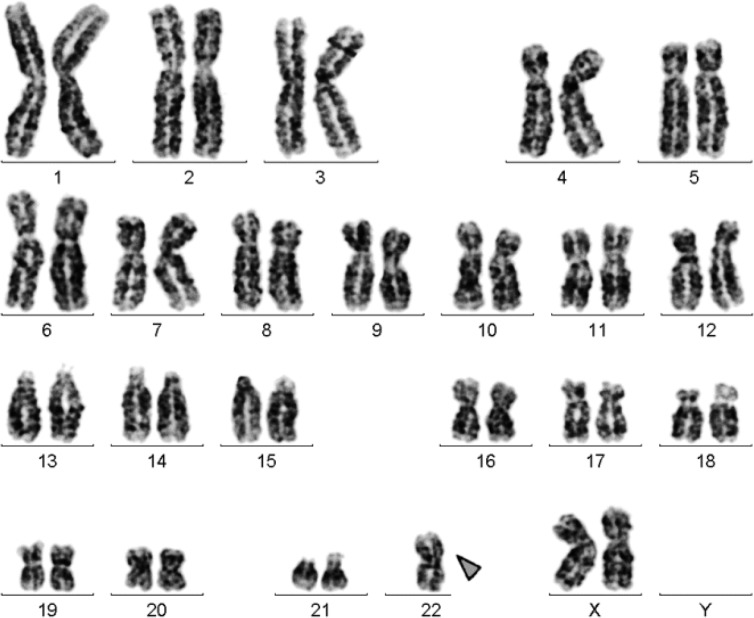
GTG-banding revealed a 45,XX, rob (22) (q10;q10). The derivative chromosome is marked by an arrowhead

**Figure 2.**
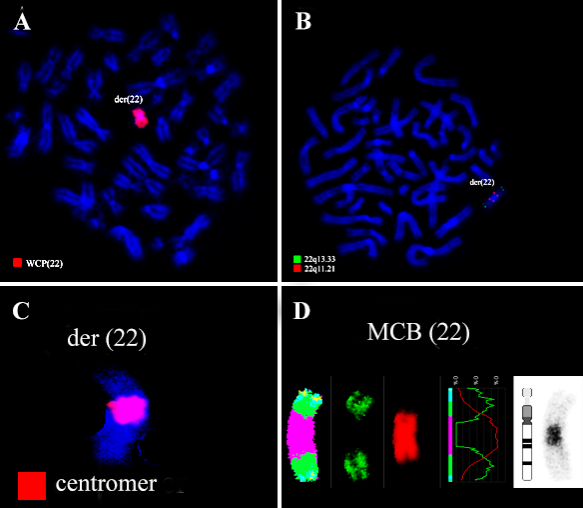
Karyotype and chromosomal aberrations were confirmed using molecular cytogenetic approaches. (A) A robertsonian translocation rob (22;22) was identified using the whole chromosome painting probe (B). Application of the probe Di-George probe revealed two red and two green signals on the derivative chromosome 22. (C) Application of all human centromer probe confirmed rob (22; 22). (D) The application of aMCB (22) confirmed rob (22;22)(q10;q10). Abbreviations: der = derivative chromosome

Banding in conventional cytogenetics revealed a karyotype of 45,XX,rob(22;22)[20] (Figure 1). This finding was further studied by molecular cytogenetics and confirmed robertsonian translocation rob (22;22) (Figure 2). Thus, the following final karyotype was determined: 45,XX,rob(22; 22)(q10;q10)[20].

**Figure 3.**
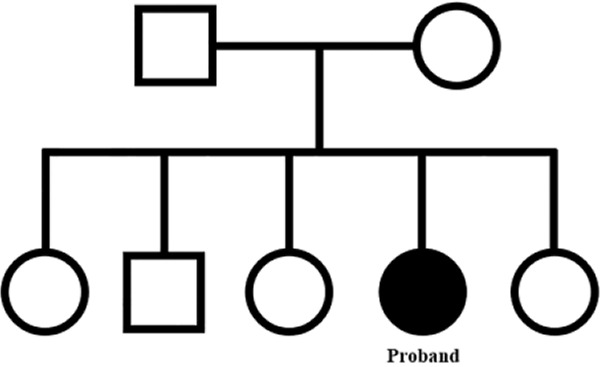
Pedigree of the proband

The karyotype of her husband was normal 46, XY. Chromosomal analysis of the phenotypically normal parents was done to ascertain the origin of abnormal chromosome. Both parents’ cytogenetics analysis revealed normal male and female karyotypes of 46,XY and 46,XX, respectively. Her brother and three sisters had normal phenotypes as well as karyotypes (46,XY and 46,XX respectively), pedigree is shown in figure 3.

## Discussion

Robertsonian translocations (ROBs) are structural chromosomal anomalies that result from the fusion of two acrocentric chromosomes (13–16, 21, 22). About 1/1000 of healthy people and 1/500 of healthy couples carry a ROB. Carriers of ROBs are often referred for reproductive counseling since they are at increased risk of spontaneous abortions, infertility and chromosomally unbalanced offsprings (12). Rob (13q14q) and rob (14q21q) are the most frequent ROBs encountered in the population (76% and 10%, respectively) (17–21). All remaining possible types of ROB constitute the remaining 15% portion of these translocations in the population.

ROBs identified in a child with an aneuploidy or through prenatal testing are more often de novo in origin than inherited from a carrier parent (22). However, rearrangements of the acrocentric chromosomes can result in nonhomologous ROB [*e.g*., rob (13q14q)] or homologous rearrangements [*e.g*., rob (21q21q)]. In nonhomologous ROBs, the breakpoints usually occur in the short arms of the participating chromosomes, resulting in dicentric translocations (23). Although the formation of a dicentric chromosome often leads to chromosome instability through anaphase bridge formation and chromosome breakage, human dicentric ROBs usually remain stable.

Homologous rearrangements of acrocentric chromosomes can result in either isochromosomes or ROBs (24). With the technological advances of molecular genetics, including the accessibility of highly polymorphic markers, homologous rearrangements can now be distinguished as isochromosomes (both arms derived from a single parental chromosome), or true ROB (translocations composed of two different, homologous chromosomes). Of all possible ROBs, 90% occur between nonhomologous chromosomes and 10% occur between homologous chromosomes (19).

Since balanced ROBs involve loss of only short arm material, carriers have normal phenotype and impaired gametogenesis (25). The fertilization with an aneuploid gamete results in monosomy or trisomy in the fetus (26). Fetal aneuploidies are a major cause of pregnancy loss (27), hence, achieving full term pregnancy is only possible if the gemmates were fertilized with suitable aneuploid gemmates which will result in Uniparental Disomy (UPD).

Early reported literatures with similar cases were all associated with RPL. Maeda et al. (28) reported a rob (22; 22) in a woman with recurrent abortions, the karyotype was determined as 46, XX, −22, +t(22q22q) and identified after cytogenetic studies of the embryonic tissue derived from one of the spontaneous abortions. Mameli et al. (29) and Granat et al. (30) reported similar two cases of rob (22;22) identified in the husband of a woman who had early RPL. In Middle East, Ocak et al. (31) observed rob (22;22)(q10;q10) in Turkish female with RPL. Also, Kiani et al. (32) reported a rob (22;22) in Iranian female case with RPL history. Both studies were performed using conventional cytogenetics methods without confirmation by molecular cytogenetic studies. The limitations of these early cases were that further molecular cytogenetic studies were not done to confirm ROB or isochromosome (31, 33). Furthermore, Zhao t al. (34) found 3 out of 872 cases had a similar rob (22:22), the results were also not confirmed by further cytogenetic studies. In the present study, the case of a female patient was reported with de novo rob (22;22)(q10;10) which involved both of short arms of chromosome 22 and this result was confirmed by molecular cytogenetics analyses.

UPD associated with an isochromosome was reported in cases with i(1p) plus i(1q), i(2p) plus i(2q), i(4p) plus i(4q), i(7p) plus i(7q), psudic (8)(p23.3), i(9p) plus i(9q), i(13q), i(14q), i(15q), i(21q), and i(22q) (35, 36). In patients with maternal and paternal UPD (22), no significant clinical impact was determined (37–39). Two early cases reported suspected UPD (22) transmission for their daughters (40–42). Later, UPD (22mat) was reported in a 25 year-old healthy man investigated following RPL in his wife (43). He had a de novo balanced rob (22q;22q) which eventually appeared to be an i(22). No additional adverse phenotypic effect appeared besides causing reproductive failure with possible monosomic or trisomic conceptions for chromosome 22 (39, 42, 43).

Gamete donation (egg or sperm), surrogacy, and adoption in many countries are methods of preventing conception of an affected embryo; it is illegal and against religious believes in the Arab world. The choice depends upon the specific abnormality and parental preference.

## Conclusion

In summary, couples with the history of RPL should be suggested to do cytogenetic analysis in order to estimate whether they have chromosomal rearrangement. However, the rob(22;22)(q10;10) is the cause of recurrent abortions. This diagnostic approach is of great significance to figure out what causes RPL. Our results may help in enforcing genetic counseling for carriers of rare ROBs.
